# Effects of cannabis oil extract on immune response gene expression in human small airway epithelial cells (HSAEpC): implications for chronic obstructive pulmonary disease (COPD)

**DOI:** 10.1186/s42238-019-0014-9

**Published:** 2020-01-31

**Authors:** Stephen W. Mamber, Volkan Gurel, Jeremy Lins, Fred Ferri, Sarah Beseme, John McMichael

**Affiliations:** 1 Beech Tree Labs Inc., 1 Virginia Ave, Suite 103, Providence, RI 02905 USA; 2 The Institute for Therapeutic Discovery, Delanson, NY 12053 USA; 3 NCM Biotechnology, Newport, RI 02840 USA

**Keywords:** HSAEpC (human small airways epithelial cells), Cannabis, Chronic obstructive pulmonary disease (COPD), Gene expression profiling, KEGG pathway analysis, Anti-inflammatory, Th1 and Th2 immune response

## Abstract

**Background:**

Chronic obstructive pulmonary disease (COPD) is commonly associated with both a pro-inflammatory and a T-helper 1 (Th1) immune response. It was hypothesized that cannabis oil extract can alleviate COPD symptoms by eliciting an anti-inflammatory Th2 immune response. Accordingly, the effects of cannabis oil extract on the expression of 84 Th2 and related immune response genes in human small airways epithelial cells (HSAEpC) were investigated.

**Methods:**

HSAEpC from a single donor were treated with three dilutions of a standardized cannabis oil extract (1:400, 1:800 and 1:1600) along with a solvent control (0.25% [2.5 ul/ml] ethanol) for 24 h. There were four replicates per treatment dilution, and six for the control. RNA isolated from cells were employed in pathway-focused quantitative polymerase chain reaction (qPCR) microarray assays.

**Results:**

The extract induced significant (*P* < 0.05) changes in expression of 37 tested genes. Six genes (CSF2, IL1RL1, IL4, IL13RA2, IL17A and PPARG) were up-regulated at all three dilutions. Another two (CCL22 and TSLP) were up-regulated while six (CLCA1, CMA1, EPX, LTB4R, MAF and PMCH) were down-regulated at the 1:400 and 1:800 dilutions. The relationship of differentially-expressed genes of interest to biologic pathways was explored using the Database for Annotation, Visualization and Integrated Discovery (DAVID).

**Conclusions:**

This exploratory investigation indicates that cannabis oil extract may affect expression of specific airway epithelial cell genes that could modulate pro-inflammatory or Th1 processes in COPD. These results provide a basis for further investigations and have prompted in vivo studies of the effects of cannabis oil extract on pulmonary function.

**Trial registration:**

NONE (all in vitro experiments).

## Background

Chronic obstructive pulmonary disease (COPD) is a respiratory ailment characterized by airway inflammation and irreversible obstruction, resulting in breathing difficulty, mucus production and coughing/wheezing, among other symptoms. As with other diseases of aging, COPD has been increasing in the global population; by 2012, COPD had become the fourth leading cause of death worldwide and is projected to become the 3rd leading cause of death in 2020 (Ferkol and Schraufnagel 2014; Gold Reports 2018). COPD and asthma, a recurring but reversible respiratory disease, share a number of common airway obstruction and inflammation symptoms. In fact, there are two competing hypotheses (termed the British hypothesis and the Dutch hypothesis) relating to the pathophysiology of these diseases (Ghebre et al. 2015). These differences in scientific consensus are addressed in part through an overlapping condition called asthma-COPD overlap syndrome, or ACOS (Gold Reports 2018; Allinson and Wedzicha 2017; Hines and Peebles 2017). ACOS provides a rationale for subsets of COPD patients with asthma-like features and vice versa (Christenson et al. 2015). That said, as distinct diseases, COPD and asthma appear to be fundamentally different from an immunological standpoint: In asthma, allergens trigger an antibody-mediated immune response via the actions of T helper 2 (Th2) cytokines such as interleukins IL-5, IL-13, IL-25 and IL-33, as well as certain Th2-related chemotactic factors. Whereas, in COPD the cumulative effects of cigarette smoke and other chemical irritants result in a pro-inflammatory cell-mediated immune response facilitated by cytokines such as IL-1 beta, IL-6, tumor necrosis factor (TNF) alpha and a variety of T helper 1 (Th1) related chemotactic factors (Barnes 2016, 2009; Schuijs et al. 2013; Barnes 2017). While there is, to date, no cure for COPD, there are various standard treatment drugs available, notably, bronchodilators and corticosteroids (Gold Reports 2018; Allinson and Wedzicha 2017; Hines and Peebles 2017; Rosenberg and Kalhan 2017). However, such treatments have variable effectiveness and often have potentially serious side effects. There have been both anecdotal reports (e.g., on the internet) and scientific studies of the use of orally-administered cannabis oil extract or other cannabinoids derived from the leaves of the marijuana plant, *Cannabis sativa*, to alleviate symptoms of COPD (Pickering et al. 2011). Cannabis oil extract is a complex mixture of substances with potential pharmacological properties (Elsohly and Slade 2005; Amin and Ali 2019). However, certain components of cannabis oil extract, such as cannabidiol, are known to have anti-inflammatory properties (Cabral et al. 2015; Klein 2005). Cannabidiol and other phytocannabinoids, such as 9-tetrahydrocannabinol, were shown to inhibit pro-inflammatory and Th1 cytokines in vitro and in in vivo models of lipopolysaccharide (LPS) induced lung injury, elicit an anti-inflammatory and Th2 immune response and potentially restore a Th1/Th2 balance in vitro, and shift the immune response profile from Th1 to Th2 in a murine model of diabetes (Petrosino et al. 2018; Ribeiro et al. 2012, 2015; Yuan et al. 2002; Weiss et al. 2006). As COPD represents a respiratory disease with a pro-inflammatory and Th1 immune response profile, the purpose of the present exploratory study was to determine if cannabis oil extract could up-regulate the in vitro expression of Th2, anti-inflammatory and related immune response genes in human small airways epithelial cells (HSAEpC). HSAEpC cells were selected as the model in vitro cell culture system for these studies in part because of the role of airway epithelial cells in respiratory system immune responses (Gras et al. 2013; Hallstrand et al. 2014; Hirota and Knight 2012; Lloyd and Saglani 2015). COPD can adversely affect immune response, host defense, cell and tissue repair and lung function in airway epithelial cells (De Rose et al. 2018). Accordingly, cannabis oil extract was tested for its effects on the expression of 84 respiratory immune response-related genes in HSAEpC using pathway-focused polymerase chain reaction (PCR) array technology. This pathway-focused array was composed of genes encoding Th2 cytokines and chemokines, cytokine and chemokine receptors, transcription factors, immune cell molecules and related proteins. Bioinformatics software was used to analyze the gene expression profiling data generated from these experiments.

## Materials and methods

### Cell culture

Human small airway epithelial cells (HSAEpC) were purchased from PromoCell (catalog number C-12642, Heidelberg, Germany). The certificate of analysis showed that the cells originated from the distal respiratory tract of a Caucasian female, age 65, frozen in passage two. (Cells were isolated in accordance with an approved tissue donation program). Each cell suspension was thawed, and cells were cultured in small airway epithelial cell growth medium (PromoCell C-21070) supplemented with growth medium supplement mix (PromoCell C-39175). Cells were grown in T-flasks at 37 C in a 5% CO_2_ incubator.

### *Cannabis sativa* oil extract

The cannabis oil extract employed in these experiments was kindly provided by F. Ferri, NCM Biotech, Newport, RI, USA. This extract, a proprietary formulation covered under US Patent 9,199,960, contained approximately 95 mg/ml of total cannabinoids in ethanol. This was composed of a mixture of about 55 mg/ml of cannabidiol plus cannabidiolic acid and about 40 mg/ml of tetrahydrocannabinol plus tetrahydrocannabinolic acid. The preparation did not contain cannabinol. An analysis for terpenes or flavonoids was not conducted; the former but not the latter was likely present in the preparation. The stock solution was diluted 1:100 in ethanol and stored at 4 C. This preparation was further diluted 1:100 into cell culture media and tested for cytotoxic effects against primary HSAEpC cells in vitro. It was found that stock extract dilutions of 1:400 and higher were not cytotoxic. Ethanol (non-cytotoxic at a 0.25% [2.5 ul/ml] final concentration) was the negative control. Neither ethanol alone nor the combination of extract in ethanol showed any cytotoxic effects at a 1:400 dilution in cell culture media. Accordingly, 1:400, 1:800 and 1:1600 stock extract dilutions (corresponding to final concentrations of 1:40,000, 1:80,000 and 1:160,000, or about 2.4, 1.2 and 0.6 μg/ml of total cannabinoids in cell culture media) were selected for further testing.

### Treatment of cells with cannabis oil extract

HSAEpC were seeded in 6-well cell culture plates at a density of 200,000 cells per well. After overnight adhesion, cells were treated for 24 h with media containing cannabis oil extract (1:400, 1:800 and 1:1600 dilutions). Negative control cells were treated with an equivalent volume of ethanol or phosphate buffered saline (PBS) (1:400 dilution for a 0.25% final concentration). Each treatment dilution had four replicates, whereas control conditions (ethanol and PBS) had 6 replicates.

### RNA extraction and reverse transcription

After 24 h of exposure to cannabis oil extract, cells were washed with PBS, collected in Trizol (Thermo Fisher Scientific, Waltham, MA) and total mRNA was extracted following manufacturer’s instructions. Briefly, samples were incubated for 5 min at room temperature and 200 ul of chloroform (Sigma-Aldrich, Saint Louis, MO) was added to 1 ml of Trizol. The aqueous phase containing RNAs was separated by centrifugation at 12,000 g for 15 min and transferred in a new tube. RNA was subsequently precipitated by adding 500 ul of isopropanol (Sigma-Aldrich) for 10 min followed by centrifugation for 10 min at 12,000 g. Pellets containing RNAs were washed three times in 75% ethanol and resuspended in 15–40 ul of nuclease-free water.

After the yield of RNA extraction was quantified using a Nanodrop (Thermo Fisher Scientific), complementary DNA (cDNA) was synthesized from 250 ng of RNA using the reverse transcription (RT2) First Strand kit (Qiagen, Valencia, CA) following the manufacturer’s instructions.

### Pathway-focused quantitative polymerase chain reaction (qPCR) assays

In order to identify respiratory-related Th2 and associated immune response genes whose expression could be altered by the cannabis oil extract treatment, RT^2^ Profiler™ PCR Arrays were used according to the manufacturer instructions (Qiagen, catalog number PAHS 067Z, product number 330231). Briefly, this assay in 384 well plate format contains primers for 84 genes of interest (GOIs) and 4 housekeeping genes in 4 replicates. A listing and description of the Th2 cytokine/chemokine and related immune response genes in this PCR array are shown in the accompanying Additional file 1: Table S1. PCR mixes including SYBR Green dye and 0.17 ul of cDNA were added to the plate wells and the PCR reaction was performed following the manufacturer’s instructions using an Applied Biosystems® ViiA™ 7 Real-Time PCR System (Thermo Fisher Scientific).

### Data, statistical and bioinformatics analyses

Quantitative PCR results were analyzed by the delta cycle threshold (Ct) method, with the 0.25% ethanol treated cells as a control, using the Data Resources Center (www.Qiagen.com). Genes with Ct values > = 35 were excluded from this analysis, and genes with Ct values > 32 were considered low expression. Fold changes (2^(− Delta Ct)) were determined as the normalized gene expression (2^(− Delta Ct)) in the cannabis extract treated sample divided by the normalized gene expression (2^(− Delta Ct)) in the control sample (ethanol treated cells). Student’s t-test was used to determine *p*-values between control group (ethanol) and each experimental group (cannabis oil extract). Genes were regarded as up-regulated or down-regulated if their extract treatment fold changes were > 2.0 or < 0.5 relative to ethanol controls and were statistically significant (*P* < 0.05). The same analysis was performed to compare the results of the 0.25% ethanol treated cells with that of the saline control cells.

Further evaluation of GOIs was accomplished using the Database for Annotation, Visualization and Integrated Discovery or DAVID program (Huang et al. 2009a, b). This software tool was selected for its ability to analyze biological relationships among a list of GOIs, including an assessment of gene expression in specific Kyoto Encyclopedia of Genes and Genomes (KEGG) pathways (Ogata et al. 1999). The Benjamini-Hochberg statistic was employed to control the false discovery rate while conducting multiple comparisons of the data (Huang et al. 2009a, b). Results from the DAVID analysis was coupled with reviews of the scientific literature regarding the prominent GOIs identified.

## Results

### Gene regulation by cannabis oil extract

**Fig. 1:**
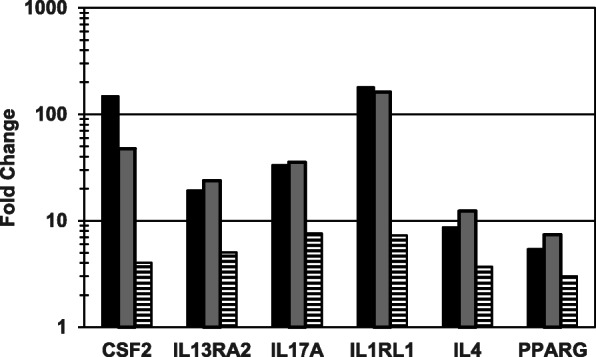
Genes of interest (GOIs) up-regulated by cannabis oil extract at all three test dilutions (dose response experiment). HSAEpC were exposed to each of three dilutions of cannabis oil extract in ethanol for 24 h. Each fold change result is based on four cannabis oil extract treatment replicates and six control replicates. Legend: Black, 1:400 dilution; Grey, 1:800 dilution; Horizontal Stripes, 1:1600 dilution

**Fig. 2:**
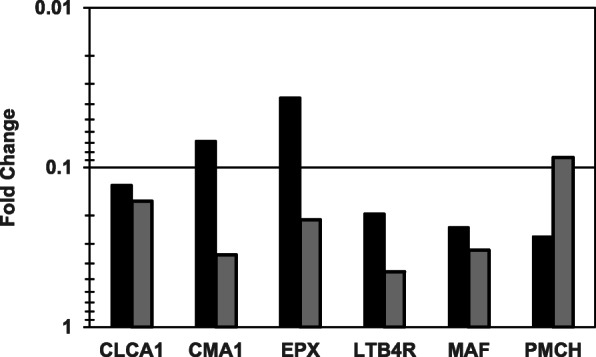
GOIs down-regulated by cannabis oil extract at the 1:400 and 1:800 test dilutions (dose response experiment). HSAEpC were exposed to each of three dilutions of cannabis oil extract in ethanol for 24 h. Each fold change result is based on four cannabis oil extract treatment replicates and six control replicates. There were no down-regulated genes at the 1:1600 dilution. Legend: Black, 1:400 dilution; Grey, 1:800 dilution

Tables 1 and 2 show qPCR fold regulation data for individual genes whose expression was significantly (*P* < 0.05) up-regulated or down-regulated, respectively, by any of the three cannabis oil extract dilutions (1:400, 1:800 and 1:1600) at 24 h. Overall, of the 84 analyzed genes, 37 (16 up-regulated and 21 down-regulated) were affected by one or more cannabis oil extract dilutions. Treatment with the 1:400 dilution resulted in 15 up-regulated and 20 down-regulated genes, while the 1:800 dilution treatment yielded nine up-regulated and seven down-regulated genes. Six genes (CSF2, IL1RL1, IL4, IL13RA2, IL17A and PPARG) were up-regulated by all three extract dilutions, with fold changes ranging from three to over 180 (Table 1 and Fig. 1). There also were two genes (CCL22 and TSLP) that were up-regulated and six genes (CLCA1, CMA1, EPX, LTB4R, MAF and PMCH) that were down-regulated at both the 1:400 and 1:800 dilutions. Fold changes for the six down-regulated genes ranged between 0.45 and < 0.04 (Table 2 and Fig. 2). Of the 47 genes that were neither up- or down-regulated, 36 showed undetectable expression in both treatment and control samples, based on Ct values > 35. No data were obtained for one of the 84 genes, IL18, because of a technical error. The 1:800 dilution of cannabis oil extract was tested two additional times under slightly modified conditions, each with its own negative control. This was done to confirm and extend the initial observations. Results were similar to that of 1:800 dilution from the primary experiment (data not shown). Finally, the ethanol control was compared to the PBS control to determine if 0.25% ethanol had an effect on gene expression. The average fold change of the 84 genes tested was 0.89, and none were significantly up-regulated. Only one gene (IL17A) was significantly down-regulated (fold change = 0.4). Interestingly, IL17A was significantly up-regulated at all three extract dilutions.

**Table 1:** Up-regulated genes of interest (GOIs) after exposure of HSAEpC to various dilutions of cannabis oil extract for 24 h

Symbol	Description	1:400 Dil.			1:800 Dil.			1:1600 Dil.		
		Fold Change	95% CI	*P*-value	Fold Change	95% CI	*P*-value	Fold Change	95% CI	*P*-value
BCL6	B-cell leukemia/lymphoma 6	2.408	(1.94, 2.88)	0.00006						
CCL22	Chemokine (C-C motif) ligand 22	5.309	(2.69, 7.93)	0.00045	5.249	(2.83, 7.67)	0.00022			
CCL24	Chemokine (C-C motif) ligand 24	59.800	(42.54, 77.06)	< 0.00001						
CCL26	Chemokine (C-C motif) ligand 26	5.396	(3.50, 7.29)	< 0.00001						
CSF2	Colony stimulating factor 2 (granulocyte-macrophage)	148.914	(118.08, 179.74)	< 0.00001	47.615	(39.28, 55.95)	< 0.00001	4.018	(3.33, 4.70)	< 0.00001
IL4	Interleukin 4	8.696	(6.26, 11.13)	0.00001	12.324	(9.57, 15.08)	< 0.00001	3.679	(2.41, 4.95)	0.00038
IL12A	Interleukin 12A (natural killer cell stimulatory factor 1, cytotoxic lymphocyte maturation factor 1, p35)	6.789	(2.15, 11.43)	0.00048						
IL12B	Interleukin 12B (natural killer cell stimulatory factor 2, cytotoxic lymphocyte maturation factor 2, p40)	5.196	(0.00001, 10.54)	0.03230						
IL13RA2	Interleukin 13 receptor, alpha 2	19.244	(16.80, 21.69)	< 0.00001	23.779	(21.19, 26.37)	< 0.00001	5.049	(4.19, 5.91)	< 0.00001
IL17A	Interleukin 17A	33.457	(21.68, 45.24)	0.00004	35.543	(25.63, 45.46)	< 0.00001	7.563	(5.64, 9.49)	< 0.00001
IL1RL1	Interleukin 1 receptor-like 1	180.497	(122.94, 238.06)	< 0.00001	161.270	(105.45, 217.09)	< 0.00001	7.296	(4.95, 9.64)	< 0.00001
PPARG	Peroxisome proliferator-activated receptor gamma	5.415	(4.81, 6.02)	< 0.00001	7.367	(6.40, 8.34)	< 0.00001	3.004	(2.63, 3.38)	< 0.00001
PRG2	Proteoglycan 2, bone marrow (natural killer cell activator, eosinophil granule major basic protein)				2.600	(2.04, 3.16)	0.00004			
PTGDR2	Prostaglandin D2 receptor 2	3.725	(1.55, 5.90)	0.00374						
TBX21	T-box 21	28.785	(23.31, 34.26)	< 0.00001						
TSLP	Thymic stromal lymphopoietin	11.999	(9.61, 14.39)	< 0.00001	2.602	(2.03, 3.18)	0.00002

**Table 2:** Down-regulated GOIs after exposure of HSAEpC to various dilutions of cannabis oil extract for 24 h

Symbol	Description	1:400 Dil.			1:800 Dil.			1:1600 Dil.		
		Fold Change	95% CI	*P*-value	Fold Change	95% CI	*P*-value	Fold Change	95% CI	*P*-value
ADRB2	Adrenergic receptor, beta 2	0.462	(0.40, 0.53)	0.00001						
AREG	Amphiregulin	0.476	(0.41, 0.54)	< 0.00001						
CCL5	Chemokine (C-C motif) ligand 5	0.226	(0.10, 0.36)	0.00119						
CLCA1	Chloride channel accessory 1	0.129	(0.03, 0.23)	0.02891	0.162	(0.06, 0.26)	0.03048			
CMA1	Chymase 1, mast cell	0.068	(0.04, 0.10)	0.00037	0.353	(0.20, 0.50)	0.00421			
EPX	Eosinophil peroxidase	0.037	(0.03, 0.04)	< 0.00001	0.213	(0.11, 0.31)	< 0.00001			
FOXP3	Forkhead box P3	0.257	(0.12, 0.40)	0.00038						
IL3RA	Interleukin 3 receptor, alpha (low affinity)				0.344	(0.16, 0.52)	0.00364			
IL4R	Interleukin 4 receptor	0.261	(0.24, 0.28)	< 0.00001						
IL5	Interleukin 5 (colony-stimulating factor, eosinophil)	0.319	(0.06, 0.58)	0.01014						
IL13RA1	Interleukin 13 receptor, alpha 1	0.343	(0.29, 0.40)	< 0.00001						
IL33	Interleukin 33	0.185	(0.16, 0.21)	< 0.00001						
LTB4R	Leukotriene B4 receptor	0.194	(0.16, 0.23)	< 0.00001	0.451	(0.39, 0.52)	0.00001			
MAF	V-maf musculoaponeurotic fibrosarcoma oncogene homolog (avian)	0.237	(0.20, 0.28)	< 0.00001	0.330	(0.29, 0.37)	0.00001			
MMP9	Matrix metallopeptidase 9 (gelatinase B, 92 kDa gelatinase, 92 kDa type IV collagenase)	0.274	(0.22, 0.33)	0.00001						
PMCH	Pro-melanin-concentrating hormone	0.271	(0.17, 0.37)	0.00188	0.087	(0.03, 0.14)	0.00047			
POSTN	Periostin, osteoblast specific factor	0.176	(0.16, 0.19)	< 0.00001						
RORC	RAR-related orphan receptor C	0.180	(0.00001, 0.36)	0.02731						
SATB1	SATB homeobox 1	0.481	(0.31, 0.65)	0.00148						
STAT5A	Signal transducer and activator of transcription 5A	0.328	(0.27, 0.38)	0.00001						
TNFSF4	Tumor necrosis factor (ligand) superfamily, member 4	0.197	(0.08, 0.31)	< 0.00001

### Use of the DAVID bioinformatics program to identify specific respiratory disease-related GOIs

In order to evaluate the biological importance of the gene expression data, the list of 35 up- and down-regulated GOIs from the 1:400 dilution treatment were further analyzed using bioinformatics software, specifically, the DAVID program. The 35 GOIs were analyzed with the Functional Annotation Tool of DAVID using the full *Homo sapiens* genome as the baseline or background control. The summarized results of this analysis, yielding a total of 33 GOIs, are shown in Table 3. The functional categories and pathways selected were based on both the numbers of GOIs within each category plus low (i.e., highly statistically significant) *p*-values and Benjamini-Hochberg correction values generated by the use of the Functional Analysis Tool. The disease component yielded 10, 22 and 17 GOIs involved in COPD, asthma and the combination category of asthma/bronchiolitis/viral (especially respiratory syncytial virus) infections, respectively. Three major functional categories were identified by DAVID enrichment analysis: Signal genes (22), cytokine genes (14), and inflammatory response genes (4). DAVID’s gene ontogeny enrichment analysis program further identified 15 and eight genes coding for gene products involved in the immune response and positive regulation of the inflammatory response, respectively. The gene ontogeny enrichment analysis for molecular functions program within DAVID identified 10 genes whose products are molecules with cytokine activity and five genes encoding molecules with cytokine receptor activity. With respect to cellular components, there were 19 and 12 GOIs, respectively, that encoded extracellular space- or extracellular region-related molecules. Additional analyses of the 35 genes using the DAVID program identified three KEGG pathways of interest. Eleven GOIs were identified in the cytokine-cytokine receptor interaction KEGG pathway. A schematic picture of how the KEGG cytokine-cytokine receptor interaction GOIs are related can be visualized at https://david.ncifcrf.gov/kegg.jsp?path=hsa04060$Cytokine-cytokine%20receptor%20interaction&termId=550028747&source=kegg. Ten regulated genes were identified in the Janus kinase and signal transducer and activator of transcription (JAK-STAT) signaling pathway.

**Table 3:** Evaluation of GOIs using the DAVID functional analytical tool

Gene Symbol	Disease-related GOI			Functional Analysis GOI			Biological Process GOI		Molecular Function GOI		Cellular Component GOI		KEGG Pathway GOI	
	COPD	Asthma	Asthma Bronchiolitis Viral/RSV	Cytokine Function	Signaling Function	Inflammatory Response Function	Immune Response	Positive Regulation of Inflammatory Response	Cytokine Activity	Cytokine Receptor Activity	Extracellular Space	Extracellular Region	Cytokine-Cytokine Receptor Interaction	Jak-STAT Signaling Pathway
ADRB2	X	X	X											
AREG				X	X				X					
BCL6	X					X		X						
CCL22			X	X	X		X	X			X	X		
CCL24		X	X	X	X	X	X	X			X			
CCL26		X	X	X	X	X	X	X			X			
CCL5	X	X	X	X	X	X	X	X			X	X	X	
CLCA1		X			X						X			
CMA1		X			X		X					X		
CSF2	X	X	X	X	X		X		X		X		X	X
EPX					X						X			
FOXP3		X	X											
IL4	X	X	X	X	X		X		X		X	X	X	X
IL4R	X	X	X		X		X			X	X		X	X
IL5		X	X	X			X	X	X		X	X	X	X
IL12A	X	X	X	X	X		X		X		X	X	X	X
IL12B	X	X	X	X	X				X	X	X	X	X	X
IL13RA1		X	X		X					X			X	X
IL13RA2			X		X					X	X	X		X
IL17A		X	X	X	X		X	X	X		X	X	X	
IL1RL1		X			X		X			X				
IL33		X		X					X		X	X		
LTB4R							X	X						
MMP9	X	X			X						X	X		
PMCH					X							X		
POSTN					X						X			
PPARG	X	X												
PTGDR2		X					X							
SATB1					X									
STAT5A			X											X
TBX21		X	X											
TNFSF4				X			X		X		X		X	
TSLP		X		X	X				X		X		X	X
GOI Count	10	22	17	14	22	4	15	8	10	5	19	12	11	10

The same bioinformatics analyses were applied specifically to the list of eight genes up-regulated by either all three dilutions or by two of three dilutions of cannabis oil extract (Table 4). Functional analysis of disease components yielded all eight GOIs in the respiratory disease category (asthma and/or bronchiolitis and/or viral infections), with two genes (IL4 and IL13RA2) specifically in the COPD/asthma category. Five GOIs (CCL22, CSF2, IL4, IL17RL1 and TSLP) were in the cytokine functional category. All GOIs except PPARG were in the signal functional category. Biological process analysis identified five GOIs (CCL22, CSF2, IL1RL1, IL4 and IL17A) with activity in the immune response category. CSF2 and IL17 were identified as positive regulators of IL-23 production, while IL4 and PPARG were identified as negative regulators of the acute inflammatory response. There were six extracellular space cell component GOIs (all but IL1RL1 and PPARG). Molecular function analysis identified CSF2, IL4, IL17A and TSLP as cytokine activity GOIs. These four were the same GOIs in the KEGG pathway for cytokine and cytokine receptor interactions. Also, there were four gene products (CSF2, IL4, IL13RA2 and TSLP) involved in the KEGG pathway for JAK-STAT signaling. Bioinformatics analysis of the six down-regulated GOIs using DAVID did not reveal common functions or KEGG pathways. However, this does not lessen the potential relevance of the individual down-regulated GOIs to the treatment of COPD.

**Table 4:** Evaluation of up-regulated GOIs using the DAVID functional analytical tool

Gene Symbol	Disease-related GOI			Functional Analysis GOI		Biological Process GOI			Molecular Function GOI	Cellular Component GOI	KEGG Pathway GOI	
	COPD/ Asthma	Asthma	Asthma Bronchiolitis Viral/RSV	Cytokine Function	Signaling Function	Immune Response	Positive Regulation of Interleukin-23 Production	Negative Regulation of Acute Inflammatory Response	Cytokine Activity	Extracellular Space	Cytokine-Cytokine Receptor Interaction	Jak-STAT Signaling Pathway
CCL22			X	X	X	X				X		
CSF2		X	X	X	X	X	X		X	X	X	X
IL1RL1		X			X	X						
IL4	X	X	X	X	X	X		X	X	X	X	X
IL13RA2	X		X		X					X		X
IL17A		X	X	X	X	X	X		X	X	X	
PPARG		X						X				
TSLP		X		X	X				X	X	X	X

## Discussion

### Experimental design considerations

These experiments were designed to determine if cannabis oil extract could in fact affect immune response gene expression in a human primary lung epithelial cell model that might be relevant to the treatment of COPD. It is acknowledged that the experimental protocols employed were exploratory in nature. Consequently, the results of this study have to be considered preliminary for a variety of reasons. These reasons include the HSAEpC being from a single individual, the time and resource constraints involved in performing these experiments, and the composition of the cannabis oil extract. That said, these results have value as a confirmation and extension of prior studies characterizing the anti-inflammatory and immune response-modifying properties of cannabinoids (Cabral et al. 2015; Klein 2005; Petrosino et al. 2018; Ribeiro et al. 2012, 2015; Yuan et al. 2002; Weiss et al. 2006). Moreover, the methodologies employed in the present study (gene expression profiling and pathway analysis) were analogous to those used in a study of the anti-inflammatory effects of cannabidiol in a multiple sclerosis model system (Kozela et al. 2016). Accordingly, these results warrant further in vitro and in vivo investigations with respect to the use of cannabis oil extract in treating COPD. In particular, these results served as the basis for testing of this extract in an animal model of pulmonary function (J. Osborn, University of Kentucky, personal communication).

### DAVID bioinformatics analysis summary

Treatment of HSAEpC cells with cannabis oil extract markedly altered expression of 37 immune response and related genes out of 84 tested after 24 h of treatment. Some of these genes are known in the literature to be associated with respiratory diseases such as COPD. Bioinformatics analysis using the DAVID program was helpful in elucidating groups of genes with related biological activities. For example, 11 GOIs were part of the same KEGG pathway for cytokine and cytokine receptor interactions. This pathway is important in the early recognition and response phases of both innate and adaptive immunity (Arakelyan et al. 2017; Gardy et al. 2009). Furthermore, out of 26 respiratory disease-related GOIs, there were 10 GOIs specifically related to COPD. This bioinformatics analysis was coupled with literature evaluations in order to highlight the most relevant of these genes. The logical starting point for this was the eight GOIs that were up-regulated at two or three test dilutions (Tables 1 and 4), along with the six GOIs that were down-regulated at two test dilutions (Table 2).

### Up-regulated GOIs

One prominent up-regulated GOI is IL4, which encodes interleukin IL-4, a known Th2 cytokine (Barczyk et al. 2006; Borish and Steinke 2003; May and Fung 2015; Röcken et al. 1996). There is already a body of research showing that increased IL-4 production can exacerbate eosinophilia and allergen-induced asthma, at least in those instances where the disease is Th2-driven. Conversely, IL-4 could modulate COPD pathophysiology in cases where COPD can be identified as a Th1-mediated inflammatory disease (Barnes 2016; Cornwell et al. 2010; Cosio et al. 2009). In one patient study, COPD severity was correlated with lower levels of IL-4, while higher IL-4 levels contributed to wound repair in the lung epithelium (Perotin et al. 2014). IL-4 also is a signaling molecule in the JAK-STAT signaling pathway (David et al. 2001). By binding to its cell surface receptor, a cytokine such as IL-4 causes receptor dimerization, mediating a signaling process involving Janus kinase phosphorylation and the subsequent phosphorylation and dimerization of STAT proteins. The activated STAT proteins in turn induce the transcription and expression of a variety of genes involved with promoting or maintaining immune responses (Yew-Booth et al. 2015). IL13RA2, which encodes IL-13 receptor alpha 2, also was up-regulated. This receptor has more complicated regulatory activities that can involve IL-4, IL-13 and their related receptors (Andrews et al. 2013, 2009; Chomarat and Banchereau 1998). In fact, while IL4 and IL13RA2 were up-regulated, IL4R (encoding IL-4 receptor) and IL13RA1 (encoding IL-13 receptor alpha 1) were downregulated at the 1:400 sample dilution. These results suggest that, despite cannabis oil extract containing a mixture of cannabinoid compounds, it was selectively regulating Th2 cytokine and cytokine receptor gene expression. The activation of the JAK-STAT signaling pathway via IL-4 or other upregulated GOIs may also promote lung epithelial cell migration and repair (Bansal et al. 2012; Crosby and Waters 2010; Kida et al. 2008). Additionally, IL-13 receptor alpha 2 has been found to inhibit the detrimental tissue remodeling induced by IL-13 in murine models of both lung inflammation and eosinophilic esophagitis, a chronic inflammation of the esophagus (Zheng et al. 2008; Zuo et al. 2010).

Both IL4 and PPARG, which encodes peroxisome proliferator-activated receptor gamma (PPAR-gamma), were identified through pathway analysis as the two GOIs whose gene products act as negative regulators of acute inflammation. PPAR-gamma is a nuclear hormone receptor with known anti-inflammatory activities (Nencioni et al. 2003; Ricote et al. 1998). There is a substantial body of literature showing that PPAR-gamma (which was up-regulated by all three cannabis oil extract dilutions) can have beneficial effects in the therapy of COPD, and more specifically, that airway epithelial cells are affected by PPAR-gamma (Belvisi and Mitchell 2009; Morissette et al. 2015; Solleti et al. 2015). PPAR-gamma was capable of both reducing cigarette smoke-induced inflammation and protecting against *Haemophilus influenzae*-induced symptom exacerbations in a mouse model of COPD (Morissette et al. 2015; Solleti et al. 2015).

The gene with the highest fold changes at all three sample dilutions was IL1RL1, which encodes IL-1 receptor-like 1 (also known as ST2). This protein is present in lung mucosa, including airway epithelial cells, and has a critical role in the Th2-mediated immune response (Coyle et al. 1999; Traister et al. 2015; Zhao and Zhao 2015). The observed levels of IL1RL1 up-regulation by all three cannabis oil extract dilutions may make this gene a suitable biomarker for further in vitro and in vivo studies.

CCL22, CCL24 and CCL26 were three chemokine genes up-regulated by cannabis oil extract. They encode chemokine (C-C motif) ligands 22, 24 and 26, respectively. While within the limits of the qPCR detection threshold, their basal expressions in PBS and ethanol controls were low, especially for CCL24, which led to a high fold change. CCL22 was the only chemokine gene of the three that was up-regulated at both the 1:400 and 1:800 dilutions. Chemokine (C-C motif) ligand 22 has been described as both a Th2-attracting and homeostatic chemokine (Ying et al. 2008; Zlotnik and Yoshie 2012). Macrophage CCL22 gene expression in COPD has been the subject of several studies. CCL22 up-regulation has been found in sputum macrophages from COPD patients (Frankenberger et al. 2011). Eapen et al. observed a reduction in M2 (Th2) macrophages and an increase in M1 (Th1, pro-inflammatory) macrophages in the small airway walls of smokers and COPD patients (Eapen et al. 2017). However, they also reported that CCL22, along with Th2-related genes IL4, IL10 and IL13 were up-regulated in bronchiolar lavage fluid from COPD patients. In the same study, mRNA levels of CCL22 was increased in the lungs of mice chronically exposed to cigarette smoke relative to normal controls. The authors noted that these chemokines and cytokines were characteristic of a Th2 (or for macrophages, M2) immune response.

Another GOI up-regulated by cannabis oil extract was TSLP, which encodes thymic stromal lymphopoietin. TSLP protein is known to be expressed in lung epithelial cells, and is associated with the Th2 immune response, including the downstream production of Th2 cytokines and chemokines such as IL-4 and chemokine (C-C motif) ligand 22 (Fang et al. 2010; He and Geha 2010; Redhu and Gounni 2012). Also, TSLP protein, in conjunction with Th2 cytokine IL-13, was capable of increasing airway epithelial cell proliferation as well as cell repair after mechanical injury (Semlali et al. 2010).

Two up-regulated genes, CSF2 and IL17A, were identified in pathway analysis as positive regulators of IL-23 production. IL-23 is a pro-inflammatory cytokine involved in the cell-mediated immune response (Oppmann et al. 2000). This cytokine has been described as heterodimeric, composed of two subunits, IL-23A and IL-12B. Neither IL23 itself or IL23A were part of the gene array, but the IL12B gene, encoding interleukin 12B (natural killer cell stimulatory factor 2, cytotoxic lymphocyte maturation factor 2, p40) was up-regulated at the 1:400 dilution. IL17A (alternatively, IL17) encodes cytokine IL-17A, which, like IL-23, plays a role in inflammation (Chen and Kolls 2017). Increased production of IL-17A can occur in both asthma and COPD, and may contribute to COPD pathophysiology (Alcorn et al. 2010; Chang et al. 2014). However, IL-17A also functions in host defense against microbial pathogens (Aujla et al. 2007; Tsai et al. 2013). Perhaps a more interesting finding was that certain T-cell populations in asthma patients can produce both IL-17A and IL-4, and such cells appear to be a combination of Th17 (an immune response involving IL-17 and separate from Th1 and Th2) and Th2 (Cosmi et al. 2010).

CSF2 (alternatively, GMCSF), the gene with the second highest fold change after IL1RL1, encodes colony stimulating factor 2 (granulocyte-macrophage), or GM-CSF. This protein, a member of the hematopoetins family of cytokines, functions in white blood cell proliferation, migration and differentiation processes (Francisco-Cruz et al. 2014; Metcalf 2008). While GM-CSF was overexpressed in submucosal and airway smooth muscle cells of both asthma and COPD patients, elevated GM-CSF expression was linked to disease severity only in asthma (Saha et al. 2009). Furthermore, the production of GM-CSF in airways epithelial cells can be induced by IL-17A, possibly with respect to protection from microbial infections (Ponce-Gallegos et al. 2017).

### Down-regulated GOIs

Pathway analysis of the six genes of interest that were significantly down-regulated by the 1:400 and 1:800 dilutions of cannabis oil extract (CLCA1, CMA1, EPX, LTB4R, MAF and PMCH) did not reveal common biological functions. That said, two of the down-regulated GOIs, CLCA1 and CMA1, appear to be highly relevant to the treatment of COPD with respect to mucus overproduction or hypersecretion. CLCA1 encodes chloride channel accessory 1, whose expression or up-regulation has been related to mucus overproduction as well as airway hyperreactivity in COPD (Hauber et al. 2005; Iwashita et al. 2012; Sala-Rabanal et al. 2015; Wang et al. 2007). The feasibility of inhibiting mucin production genes as a viable approach to treating COPD has been postulated (Ha and Rogers 2016). Accordingly, the use of cannabis oil extract to inhibit the expression of mucin production genes such as CLCA1 could help in controlling airway mucus hypersecretion. CMA1 encodes mast cell chymase 1, a serine proteinase. Chymase can stimulate mucin secretion from lung epithelial cells, and is known to contribute to the mucus hypersecretion process in patients with COPD (He and Zheng 2004). Also, mast cell chymase and other serine proteases are known to promote inflammation through degradation of the extracellular matrix (de Garavilla et al. 2005). An inhibitor of these proteases had anti-inflammatory effects that could be related to the treatment of COPD. The down-regulation of CMA1 would therefore potentially decrease both mucus hypersecretion and inflammation.

Two other GOIs down-regulated by cannabis oil extract that may have relevance to COPD were LTB4R (alternatively, BLT1 or LTB4R1), which encodes leukotriene B4 receptor, and EPX, which encodes eosinophil peroxidase. Leukotriene B4 is an arachidonic acid-derived, neutrophil recruiting, pro-inflammatory lipid molecule, and both this leukotriene and its receptor have a role in the pathophysiology of COPD (Dong et al. 2016; Marian et al. 2006; Pace et al. 2013). The targeting of the B4 receptor has been considered as a viable approach to the treatment of COPD (Grönke et al. 2008; Hicks et al. 2010). Accordingly, the down-regulation of LTB4R by cannabis oil extract appears to be yet another indication of its anti-inflammatory potential. Interestingly, PPAR-gamma, which was up-regulated by cannabis oil extract, can also counteract inflammation mediated by leukotriene B4 in COPD (Yin et al. 2014). While eosinophil peroxidase does not appear to have been investigated as target for the treatment of COPD, it has been identified as a biomarker of the disease (Nair et al. 2013; Yang et al. 2017).

The remaining two genes down-regulated at the 1:400 and 1:800 test dilutions were MAF, encoding V-maf musculoaponeurotic fibrosarcoma oncogene homolog (avian), and PMCH, encoding Pro-melanin-concentrating hormone. Neither of these genes appears to have an obvious role in COPD, based on a literature search. It is interesting that a substantial number of the down-regulated GOIs encode either enzymes or proteins with functions other than as immune signaling cytokines or chemokines. There were also a number of GOIs that were up- or down-regulated at a single dilution that may prove relevant to the treatment of COPD by cannabis oil extract. Perhaps the most notable of these genes was MMP9, which encodes matrix metallopeptidase 9 (gelatinase B, 92 kDa gelatinase, 92 kDa type IV collagenase). This extracellular matrix-degrading metallopeptidase functions in a variety of normal physiological processes. However, expression and activation of MMP-9 has been associated with the pathophysiology of COPD (Grzela et al. 2016; Navratilova et al. 2016; Papakonstantinou et al. 2015). Interestingly, Zhou et al. reported that the concentration of MMP-9 was increased while that of PPAR-gamma was decreased in sputum samples from COPD patients, adversely affecting respiratory function (Zhou et al. 2017). This research indicated that PPAR-gamma had a protective effect in preventing an imbalance between MMP-9 and TIMP-1, a metalloproteinase inhibitor.

### Comparisons with other studies

The results of this study were consistent with other investigations demonstrating that *cannabis*-derived natural products (e.g., cannabidiol and tetrahydrocannabinol) have anti-inflammatory properties and/or are capable of generating a Th2 immune response (Cabral et al. 2015; Klein 2005; Petrosino et al. 2018; Ribeiro et al. 2012, 2015; Yuan et al. 2002; Weiss et al. 2006). IL4 (encoding IL-4) and PPARG (encoding PPAR-gamma) were two of the prominent GOIs or gene products in common between these studies and the present investigation (Klein 2005; Yuan et al. 2002; Weiss et al. 2006). The up-regulation of IL17, which was noted to be somewhat paradoxical, was also reported in a study of the effects of cannabidiol on immune response in zebrafish (Jensen et al. 2018). Otherwise, although the majority of up- and down-regulated GOIs from the present study are relevant to COPD, their regulation may represent novel findings with respect to cannabinoid effects. In contrast to the present in vitro studies, Vuolo et al. reported that cannabidiol’s anti-inflammatory activity in a mouse model of asthma was from the inhibition of both Th1 and Th2 cytokines (Vuolo et al. 2015). It is speculated that the mixture of compounds in the cannabis oil extract used in these studies may have been more capable of eliciting a Th2 immune response than purified cannabidiol. In support of this, a mixture containing both cannabidiol and tetrahydrocannabinol promoted a Th2 and anti-inflammatory immune response in a murine experimental autoimmune encephalomyelitis model (Al-Ghezi et al. 2019). It was noted that neither cannabidiol nor tetrahydrocannabinol alone was capable of eliciting the beneficial immune response.

## Conclusion

The data from these exploratory experiments suggest that treatment of HSAEpC cells with cannabis oil extract results in regulation of specific genes relevant to COPD. Cannabis oil extract significantly modulated the expression of 11 genes within a specific KEGG pathway (cytokine-cytokine receptor interactions). The expression of 10 genes in the KEGG pathway for JAK-STAT signaling also were modulated by the extract. Most of the up-regulated genes, such as IL4, CCL22 and TSLP, encode cytokines and chemokines that are involved in both anti-inflammatory and Th2-type immune responses. There were also certain GOIs, such as CLCA1 and CMA1, whose down-regulation could have beneficial effects in the treatment of COPD. In short, these preliminary results suggest that cannabis oil extract has the potential to help decrease inflammation, restore a Th1/Th2 balance, or ameliorate other symptoms in COPD, and possibly do the same in autoimmune or other diseases that have a hyperactive Th1-mediated immune response profile. On the other hand, the data suggest that cannabis oil extract would be contraindicated in diseases that are characterized by an aberrant Th2 immune response profile, such as asthma and allergy. It also would not be useful for subsets of COPD patients with asthma-like features, e.g., ACOS (Gold Reports 2018; Allinson and Wedzicha 2017; Hines and Peebles 2017). As a final note, the results of this study, which employed a preparation intended for oral delivery of cannabinoids, likely would not apply to ingestion of cannabinoids via smoking, which is deleterious to COPD patients. That said, based on these in vitro results, further investigations of cannabis oil extract for the treatment of COPD or other Th1-based diseases is warranted. In addition to further experiments in various in vitro model systems to confirm and extend these observations, in vivo experiments can be conducted to determine if cannabis oil extract has beneficial effects on pulmonary function in animal models of COPD. Subsequent to the results of the present study, this extract was tested for its effects on pulmonary function in Caribbean Vervets (*Chlorocebus aethiops sabaeus*). The in vivo data indicated that cannabis oil extract improved inspiratory lung functions (J. Osborn, University of Kentucky, manuscript in preparation). These experiments provide further evidence of the potential utility of cannabis oil extract in the treatment of COPD.

## Supplementary information

**Additional file 1:** **Table S1.** Symbols and descriptions of respiratory genes used in pathway-focused PCR arrays.

## Data Availability

As per attached.

## Abbreviations

ACOS: Asthma-COPD Overlap Syndrome
cDNA: Complementary DNA
COPD: Chronic Obstructive Pulmonary Disease
Ct: Cycle Threshold
DAVID: Database for Annotation, Visualization and Integrated Discovery
DNA: Deoxyribonucleic Acid
GOI: Gene(s) of Interest
HSAEpC: Human Small Airways Epithelial Cells
IL: Interleukin
JAK-STAT: Janus Kinase and Signal Transducer and Activator of Transcription
KEGG: Kyoto Encyclopedia of Genes and Genomes
PBS: Phosphate-buffered Saline
PCR: Polymerase Chain Reaction
qPCR: Quantitative Polymerase Chain Reaction
RNA: Ribonucleic Acid
RT: Reverse Transcription
Th1: T-helper 1
Th2: T-helper 2
TNF: Tumor Necrosis Factor
