# Supplementary material for: Effects of cannabis oil extract on immune response gene expression in human small airway epithelial cells (HSAEpC): implications for chronic obstructive pulmonary disease (COPD)
Source: J Cannabis Res. 2020 Jan 31;2:5. doi: 10.1186/s42238-019-0014-9 (PMC7819312; doi:10.1186/s42238-019-0014-9)
Supplement: Supplementary file 1 — Additional file 1: Table S1. Symbols and descriptions of respiratory genes used in pathway-focused PCR arrays. [file 42238_2019_14_MOESM1_ESM.docx]

**Table S1. Symbols and descriptions of respiratory (allergy and asthma) genes used in pathway-focused PCR arrays.**

| **Symbol** | **Description** | **Unigene^1^** | **Refseq^2^** |
| --- | --- | --- | --- |
| ADAM33 | ADAM metallopeptidase domain 33 | Hs.173716 | NM_025220 |
| ADRB2 | Adrenergic, beta-2-, receptor, surface | Hs.2551 | NM_000024 |
| ALOX5 | Arachidonate 5-lipoxygenase | Hs.89499 | NM_000698 |
| AREG | Amphiregulin | Hs.645475 | NM_001657 |
| ARG1 | Arginase, liver | Hs.440934 | NM_000045 |
| BCL6 | B-cell CLL/lymphoma 6 | Hs.478588 | NM_001706 |
| CCL11 | Chemokine (C-C motif) ligand 11 | Hs.54460 | NM_002986 |
| CCL17 | Chemokine (C-C motif) ligand 17 | Hs.546294 | NM_002987 |
| CCL2 | Chemokine (C-C motif) ligand 2 | Hs.303649 | NM_002982 |
| CCL22 | Chemokine (C-C motif) ligand 22 | Hs.534347 | NM_002990 |
| CCL24 | Chemokine (C-C motif) ligand 24 | Hs.247838 | NM_002991 |
| CCL26 | Chemokine (C-C motif) ligand 26 | Hs.131342 | NM_006072 |
| CCL5 | Chemokine (C-C motif) ligand 5 | Hs.514821 | NM_002985 |
| CCL8 | Chemokine (C-C motif) ligand 8 | Hs.271387 | NM_005623 |
| CCR3 | Chemokine (C-C motif) receptor 3 | Hs.506190 | NM_001837 |
| CCR4 | Chemokine (C-C motif) receptor 4 | Hs.184926 | NM_005508 |
| CCR8 | Chemokine (C-C motif) receptor 8 | Hs.113222 | NM_005201 |
| CD40LG | CD40 ligand | Hs.592244 | NM_000074 |
| CHI3L1 | Chitinase 3-like 1 (cartilage glycoprotein-39) | Hs.382202 | NM_001276 |
| CHIA | Chitinase, acidic | Hs.128814 | NM_201653 |
| CLC | Charcot-Leyden crystal protein | Hs.889 | NM_001828 |
| CLCA1 | Chloride channel accessory 1 | Hs.194659 | NM_001285 |
| CMA1 | Chymase 1, mast cell | Hs.135626 | NM_001836 |
| CPA3 | Carboxypeptidase A3 (mast cell) | Hs.646 | NM_001870 |
| CRLF2 | Cytokine receptor-like factor 2 | Hs.287729 | NM_001012288 |
| CSF2 | Colony stimulating factor 2 (granulocyte-macrophage) | Hs.1349 | NM_000758 |
| CSF3R | Colony stimulating factor 3 receptor (granulocyte) | Hs.524517 | NM_000760 |
| CYSLTR1 | Cysteinyl leukotriene receptor 1 | Hs.733809 | NM_006639 |
| EPX | Eosinophil peroxidase | Hs.279259 | NM_000502 |
| FCER1A | Fc fragment of IgE, high affinity I, receptor for; alpha polypeptide | Hs.897 | NM_002001 |
| FOXP3 | Forkhead box P3 | Hs.247700 | NM_014009 |
| GATA3 | GATA binding protein 3 | Hs.524134 | NM_002051 |
| PTGDR2 | Prostaglandin D2 receptor 2 | Hs.299567 | NM_004778 |
| ICOS | Inducible T-cell co-stimulator | Hs.56247 | NM_012092 |
| IFNG | Interferon, gamma | Hs.856 | NM_000619 |
| IFNGR2 | Interferon gamma receptor 2 (interferon gamma transducer 1) | Hs.634632 | NM_005534 |
| IL10 | Interleukin 10 | Hs.193717 | NM_000572 |
| IL12A | Interleukin 12A (natural killer cell stimulatory factor 1, cytotoxic lymphocyte maturation factor 1, p35) | Hs.673 | NM_000882 |
| IL12B | Interleukin 12B (natural killer cell stimulatory factor 2, cytotoxic lymphocyte maturation factor 2, p40) | Hs.674 | NM_002187 |
| IL13 | Interleukin 13 | Hs.845 | NM_002188 |
| IL13RA1 | Interleukin 13 receptor, alpha 1 | Hs.496646 | NM_001560 |
| IL13RA2 | Interleukin 13 receptor, alpha 2 | Hs.336046 | NM_000640 |
| IL17A | Interleukin 17A | Hs.41724 | NM_002190 |
| IL17RB | Interleukin 17 receptor B | Hs.654970 | NM_018725 |
| IL18 | Interleukin 18 (interferon-gamma-inducing factor) | Hs.83077 | NM_001562 |
| IL1RL1 | Interleukin 1 receptor-like 1 | Hs.66 | NM_016232 |
| IL21 | Interleukin 21 | Hs.567559 | NM_021803 |
| IL25 | Interleukin 25 | Hs.302036 | NM_022789 |
| IL2RA | Interleukin 2 receptor, alpha | Hs.231367 | NM_000417 |
| IL3 | Interleukin 3 (colony-stimulating factor, multiple) | Hs.694 | NM_000588 |
| IL31 | Interleukin 31 | Hs.569071 | NM_001014336 |
| IL33 | Interleukin 33 | Hs.731660 | NM_033439 |
| IL3RA | Interleukin 3 receptor, alpha (low affinity) | Hs.632790 | NM_002183 |
| IL4 | Interleukin 4 | Hs.73917 | NM_000589 |
| IL4R | Interleukin 4 receptor | Hs.513457 | NM_000418 |
| IL5 | Interleukin 5 (colony-stimulating factor, eosinophil) | Hs.2247 | NM_000879 |
| IL5RA | Interleukin 5 receptor, alpha | Hs.68876 | NM_000564 |
| IL9 | Interleukin 9 | Hs.960 | NM_000590 |
| KIT | V-kit Hardy-Zuckerman 4 feline sarcoma viral oncogene homolog | Hs.479754 | NM_000222 |
| KITLG | KIT ligand | Hs.661108 | NM_003994 |
| LTB4R | Leukotriene B4 receptor | Hs.567248 | NM_181657 |
| MAF | V-maf musculoaponeurotic fibrosarcoma oncogene homolog (avian) | Hs.134859 | NM_005360 |
| MMP9 | Matrix metallopeptidase 9 (gelatinase B, 92kDa gelatinase, 92kDa type IV collagenase) | Hs.297413 | NM_004994 |
| MRC1 | Mannose receptor, C type 1 | Hs.75182 | NM_002438 |
| MS4A2 | Membrane-spanning 4-domains, subfamily A, member 2 (Fc fragment of IgE, high affinity I, receptor for; beta polypeptide) | Hs.386748 | NM_000139 |
| PDCD1 | Programmed cell death 1 | Hs.158297 | NM_005018 |
| PMCH | Pro-melanin-concentrating hormone | Hs.707990 | NM_002674 |
| POSTN | Periostin, osteoblast specific factor | Hs.136348 | NM_006475 |
| PPARG | Peroxisome proliferator-activated receptor gamma | Hs.162646 | NM_015869 |
| PRG2 | Proteoglycan 2, bone marrow (natural killer cell activator, eosinophil granule major basic protein) | Hs.512633 | NM_002728 |
| RETNLB | Resistin like beta | Hs.307047 | NM_032579 |
| RNASE2 | Ribonuclease, RNase A family, 2 (liver, eosinophil-derived neurotoxin) | Hs.728 | NM_002934 |
| RNASE3 | Ribonuclease, RNase A family, 3 | Hs.73839 | NM_002935 |
| RORC | RAR-related orphan receptor C | Hs.256022 | NM_005060 |
| SATB1 | SATB homeobox 1 | Hs.517717 | NM_002971 |
| SIGLEC8 | Sialic acid binding Ig-like lectin 8 | Hs.447899 | NM_014442 |
| STAT5A | Signal transducer and activator of transcription 5A | Hs.437058 | NM_003152 |
| STAT6 | Signal transducer and activator of transcription 6, interleukin-4 induced | Hs.524518 | NM_003153 |
| TBX21 | T-box 21 | Hs.272409 | NM_013351 |
| TGFB1 | Transforming growth factor, beta 1 | Hs.645227 | NM_000660 |
| TNFRSF4 | Tumor necrosis factor receptor superfamily, member 4 | Hs.129780 | NM_003327 |
| TNFSF4 | Tumor necrosis factor (ligand) superfamily, member 4 | Hs.181097 | NM_003326 |
| TPSAB1 | Tryptase alpha/beta 1 | Hs.592982 | NM_003294 |
| TSLP | Thymic stromal lymphopoietin | Hs.389874 | NM_033035 |

1. Gene identification number from the National Center for Biotechnology Information (NCBI) database of transcriptomes.
2. Gene identification number from the NCBI database of nucleotide sequences.
